# Early Post-ischemic Brain Glucose Metabolism Is Dependent on Function of TLR2: a Study Using [^18^F]F-FDG PET-CT in a Mouse Model of Cardiac Arrest and Cardiopulmonary Resuscitation

**DOI:** 10.1007/s11307-021-01677-y

**Published:** 2021-11-15

**Authors:** Rika Bajorat, Jens Kurth, Jan Stenzel, Brigitte Vollmar, Bernd J. Krause, Daniel A. Reuter, Tobias Schuerholz, Stefan Bergt

**Affiliations:** 1 Department of Anesthesiology and Intensive Care Medicine, Rostock University Medical Centre, Schillingallee 35, 18057 Rostock, Germany; 2 Department of Nuclear Medicine, Rostock University Medical Centre, Rostock, Germany; 3 Core Facility “Multimodale Kleintierbildgebung”, Rostock University Medical Centre, Rostock, Germany; 4 Institute of Experimental Surgery, Rostock University Medical Centre, Rostock, Germany

**Keywords:** Cardiac arrest, Resuscitation, Brain glucose metabolism, Cerebral injury, [^18^F]F-FDG, PET-CT, TLR2, Brain imaging

## Abstract

**Purpose:**

The mammalian brain glucose metabolism is tightly and sensitively regulated. An ischemic brain injury caused by cardiac arrest (CA) and cardiopulmonary resuscitation (CPR) affects cerebral function and presumably also glucose metabolism. The majority of patients who survive CA suffer from cognitive deficits and physical disabilities. Toll-like receptor 2 (TLR2) plays a crucial role in inflammatory response in ischemia and reperfusion (I/R). Since deficiency of TLR2 was associated with increased survival after CA-CPR, in this study, glucose metabolism was measured using non-invasive [^18^F]F-FDG PET-CT imaging before and early after CA-CPR in a mouse model comparing wild-type (WT) and TLR2-deficient (TLR2^−/−^) mice. The investigation will evaluate whether FDG-PET could be useful as an additional methodology in assessing prognosis.

**Procedures:**

Two PET-CT scans using 2-deoxy-2-[^18^F]fluoro-D-glucose ([^18^F]F-FDG) tracer were carried out to measure dynamic glucose metabolism before and early after CPR. To achieve this, anesthetized and ventilated adult female WT and TLR2^−/−^ mice were scanned in PET-CT. After recovery from the baseline scan, the same animals underwent 10-min KCL-induced CA followed by CPR. Approximately 90 min after CA, measurements of [^18^F]F-FDG uptake for 60 min were started. The [^18^F]F-FDG standardized uptake values (SUVs) were calculated using PMOD-Software on fused FDG-PET-CT images with the included 3D Mirrione-Mouse-Brain-Atlas.

**Results:**

The absolute SUV_mean_ of glucose in the whole brain of WT mice was increased about 25.6% after CA-CPR. In contrast, the absolute glucose SUV in the whole brain of TLR2^−/−^ mice was not significantly different between baseline and measurements post CA-CPR. In comparison, baseline measurements of both mouse strains show a highly significant difference with regard to the absolute glucose SUV in the whole brain. Values of TLR2^−/−^ mice revealed a 34.6% higher glucose uptake.

**Conclusions:**

The altered mouse strains presented a different pattern in glucose uptake under normal and ischemic conditions, whereby the post-ischemic differences in glucose metabolism were associated with the function of key immune factor TLR2. There is evidence for using early FDG-PET-CT as an additional diagnostic tool after resuscitation. Further studies are needed to use PET-CT in predicting neurological outcomes.

## Introduction

Out-of-hospital cardiac arrest (CA) strikes every year about 95.9/100,000 adults worldwide [1]. Prognosis remains very poor: European data shows in-hospital mortality of nearly 90% of patients [2, 3]. And even in survivors in particular neurological prognosis is very limited. In consequence, only less than 10% of patients return to a self-controlled life [4, 5]. The major mechanism for cerebral damage is of course the circulatory arrest that directly leads to a lack of oxygenation of brain tissue [6]. Persistent hypoxemia further aggravates global cerebral ischemia and the induced neuronal cell damage [7]. Although cardiopulmonary resuscitation (CPR) and return of spontaneous circulation (ROSC) lead to cerebral reperfusion and oxygenation, a further consequence is a severe ischemia–reperfusion (I/R) injury leading to an excessive systemic inflammatory response, often called post-cardiac arrest syndrome [8–10]. Cerebral inflammation is characterized by activation of glial cells, an influx of peripheral immune and inflammatory cells, high concentrations of reactive oxygen species (ROS), and release of pro-inflammatory mediators such as cytokines and adhesion molecules [11–14]. Toll-like receptors (TLRs) are an integral part of the innate immune response in many pathologies. In particular, TLR2 plays a central role in the activation of inflammatory response under I/R. It initiates downstream signal pathways to induce the release of pro-inflammatory cytokines (e.g., TNF-α, IL-1β), enzymes such as iNOS, and adhesion molecules as ICAM-1 [15, 16]. Furthermore, TLR2 mediates crosstalk between the cellular and humoral innate immune response [17]. So, TLR2 is also involved in the immunological response to ischemic brain injury [18–23]. Interestingly, TLR2-deficient (TLR2^−/−^) individuals showed lower release of pro-inflammatory cytokines, which improved survival after CA-CPR in the mouse model [24].

The human brain consumes about one-fifth of the whole body’s glucose as its primary source of energy with very complex regulatory mechanisms [25, 26]. Monitoring cerebral glucose metabolism therefore might enable to add functional information on the extent of cerebral damage, its recovery, and potentially also assessment of neurological prognosis. Positron emission tomography with computed tomography (PET-CT) with the use of 2-deoxy-2-[^18^F]fluoro-D-glucose ([^18^F]F-FDG) as tracer allows to investigate cerebral glucose metabolism. There are stimulating study results using this method in different models of neuronal diseases, and also in hypoxic-ischemic and traumatic brain injury [27–31]. But so far, there are only sparse data regarding cardiac arrest brain injury [32, 33].

Therefore, we investigated brain glucose metabolism with [^18^F]F-FDG PET-CT in wild-type (WT) and TLR2-deficient (TLR2^−/−^) mice before induction of CA (baseline metabolism) and early after (post) CA-CPR.

## Materials and Methods

### Animals

Wild-type (WT, C57BL/6 J, *n* = 14) and TLR2-deficient (TLR2^−/−^, B6.129-Tlr2^tm1Kir^/J, *n* = 13) 4–5-month-old mice, both female, were used with a bodyweight of approximately 20 g each. Animals were housed in a temperature-controlled environment (22 °C) under a 12:12-h dark/light cycle with free access to water and food (approved by the Ethical Committee for Care and Use of Laboratory Animals, permission number: LALLF M-V/TDS/7221.3–1-068/15). For measurements of cytokines and signaling molecules, native serum samples from seven WT mice without any intervention were used as a native control (permission number: LALLF 7221.3–1.1–022/11).

### Study Groups and Experimental Protocol

**Fig. 1.**
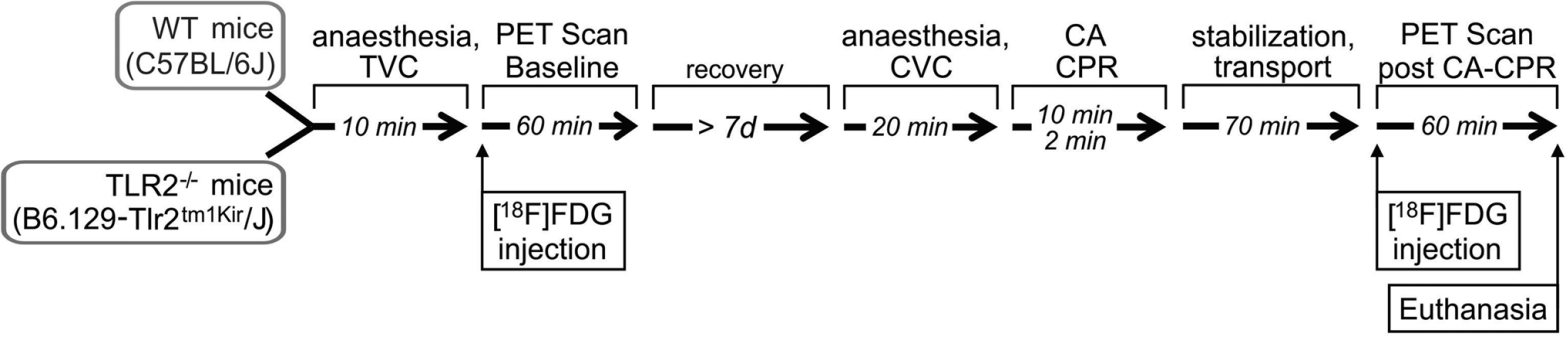
Study protocol and timeline of experimental procedure. [^18^F]F-FDG—2-deoxy-2-[^18^F]fluoro-D-glucose, CA cardiac arrest, CPR cardiopulmonary resuscitation, CVC central venous catheter, d days, min minutes, PET positron emission tomography, TLR Toll-like receptor, TVC tail venous catheter, WT wild type.

Two groups of mice, WT- and TLR2^−/−^ mice, were studied. Each group consisted of ten animals for analysis. The experimental protocol, which is outlined in detail in Fig. 1, envisaged baseline PET imaging, recovery from anesthesia for at least 7 days, followed by a standardized model of cardiac arrest and resuscitation, and then followed by a post-intervention PET imaging. During the whole experimental time, the animals had free access to water and food. No fasting protocol was used because the model of resuscitation should be as close as possible to the emergency of sudden CA, and hypoglycemia prior to CA appears to be predictive for a poor cardiac outcome [34]. PET-CT measurements were performed at the same daytime, started between 9 am and 2 pm. Blood plasma samples were collected immediately after completion of the second PET-CT.

### Anesthesia

All interventions (baseline PET imaging, cardiac arrest and resuscitation, post-intervention PET imaging) were performed under general anesthesia. Mice were anaesthetized by intraperitoneal injection of 12 µg/g ketamine and 8 µg/g xylazine. A custom-made micro catheter for the [^18^F]F-FDG injection was placed into the tail vein (baseline) and animals were then immediately intubated employing a 22-gauge cannula. Mechanical ventilation was initiated with fraction of inspired oxygen (FiO_2_) of 0.4, a tidal volume of 10 µl/g, and a respiratory rate of 120 breaths per minute.

### Positron Emission Tomography Imaging

Baseline and post-intervention [^18^F]F-FDG PET studies were performed in a small animal PET-CT (Siemens Inveon PET/CT, Siemens Healthineers, Erlangen). Animals together with the ventilator were carefully transferred to the PET bed and fixed. The acquisition of 60-min dynamic PET as list mode data set was started immediately before the injection of [^18^F]F-FDG (2-deoxy-2-[^18^F]fluoro-D-glucose) in 0.2 ml normal saline (Table 1), which was injected via a tail vein catheter at baseline and post-intervention via a central venous catheter.

**Table 1.** Hemodynamic and physical parameters before and after CA-CPR and injected amount of tracer [^18^F]F-FDG and glucose level. Data shown as mean ± SEM. No significant difference was seen between experimental groups (Mann–Whitney *U* test) or between baseline measurements and post CA-CPR (Wilcoxon signed-rank test). Injected quantity of [^18^F]F-FDG tracer was almost the same for both groups and time points. ^$^ In each row, there are the values which served for the statistical comparison, see *p* value in the last column. This required repetitions in the comparison of baseline measurements between mouse strains

Parameter	Experimental groups in PET imaging				*p*-values
	WT mice (*n* = 10)		TLR2^−/−^ mice (*n* = 10)		
	Baseline PET-CT	PET-CT post CA-CPR	Baseline PET-CT	PET-CT post CA-CPR	
Before CA-CPR					
Heart rate (1/min)		222 ± 4		214 ± 9	0.136
MAP (mm Hg)		71.67 ± 4.79		80.75 ± 6.69	0.308
Body temperature (°C)		35.89 ± 0.06		35.94 ± 0.05	0.932
CPR					
ROSC time (s)		66.5 ± 8.6		72.5 ± 8.7	0.356
ROSC rate		100%		100%	
Epinephrine (μg)		12.5 ± 1.12		13.5 ± 1.5	0.46
1 h post CA-CPR					
Heart rate (1/min)		374 ± 33		311 ± 20	0.452
MAP (mm Hg)		48.33 ± 1.67		58.33 ± 6.01	0.518
Body temperature (°C)		36.280.08		36.22 ± 0.10	0.564
Mean	16.65 ± 0.27	16.77 ± 0.67			0.878
[^18^F]F-FDG (MBq) ^$^			17.44 ± 0.65	15.78 ± 0.51	0.398
	16.65 ± 0.27		17.44 ± 0.65		0.481
Range (MBq)	15.14–17.97	13.72–20.36	15.02–22.01	13.99–18.65	
Glucose (mmol/l)		15.79 ± 7.29		17.73 ± 4.87	0.505

All PET studies were reconstructed as series 3D PET images of multiple frames with various time durations (15 × 20 s; 10 × 60 s; 9 × 300 s) using a 2D-ordered subsets expectation maximization algorithm (four iterations, six subsets) resulting in a voxel size of 0.86 × 0.86 × 0.79 mm. Whole-body CT scan was used for attenuation correction and PET studies were also corrected for random coincidences, dead time, scatter, and decay.

### Cardiac Arrest and Resuscitation

The model of CA-CPR was conducted as described previously [24, 35]. Briefly, CA was induced by injection of 80 µg/g potassium chloride via a central venous catheter inserted into the right jugular vein, and mechanical ventilation was interrupted. Resuscitation was initiated following 10 min of CA, precordial chest compressions begun with a frequency of 450/min, 0.4 µg/g epinephrine was injected, and ventilation was resumed (220/min; FiO_2_ 1.0). After 2 min of CPR, respiratory rate was reduced to 120/min, FiO_2_ to 0.6, and turned to FiO_2_ 0.4 after 20 min of successful resuscitation. Following ROSC, all animals received 200 µl of isotonic saline to prevent dehydration. One hour after ROSC, the post-intervention PET studies were performed as described above.

### Image Analysis

Image analysis was performed using PMOD v3.7 (PMOD Technologies LLC, Zurich, Switzerland). For standardized delineation of the target regions, the implemented T2-weighted mouse brain MR template by Mirrione et al. was used [36, 37]. The animal-specific CT datasets were spatially normalized to the MRI dataset of this atlas. The respective transformation matrices were used to also normalize the PET datasets into the Mirrione matrix. All transformations were performed using a rigid matching algorithm as implemented in PMOD. The predefined region VOIs of the Mirrione atlas were used to extract time-activity curves (TAC) from the dynamic PET data. To also determine the glucose uptake in the late phase (static) in the defined brain regions, the last three frames of the dynamic data set (15 min) were averaged. The uptake values were presented as mean standardized uptake value (SUV_mean_) and were obtained by normalizing tissue radioactivity concentration to injected dose and bodyweight of the specific animal.

### Analysis of Blood Plasma Samples

Glucose was measured from plasma samples using Bayer’s ContourXT with Contour next sensors for blood glucose determination (Ascensia Diabetes Care DeutschlandAG, Leverkusen, Germany). The concentration of glucose was given in mmol/l.

To assess early inflammatory processes, the plasma cytokines interleukin-6 (IL-6), interleukin 1β (lL-1β), tumor necrosis factor α (TNFα), and the signal molecule vascular endothelial growth factor A (VEGF-A) were measured using electrochemiluminescence-based assays from Meso Scale Diagnostics (MSD, Rockville, MD, USA). Therefore, a U-PLEX assay was used according to the manufacturer’s recommendations and all samples were analyzed in duplicates. Analyses were done using the MESO QuickPlex SQ 120 instrument (MSD) and DISCOVERY WORKBENCH® 4.0 software (MSD). For the purposes of statistical analyses, any value that was below the lowest limit of detection (LLOD) for the assay was considered negative and assigned a value of 0 pg/ml.

### Statistics

Results are presented as boxplots showing the quartiles, the 5th and 95th percentiles (whiskers), the median (line) and the mean (x), or mean ± standard deviation (SD). Differences in glucose uptake were assessed and significance was tested using Wilcoxon signed-rank test for related and Mann–Whitney *U* test for independent samples (SPSS 22). With respect to significance, we first set the level of significance to *p* ≤ 0.05. To account for multiple testing, Bonferroni correction was used. On the basis of the known variance of individual experiments, the effect sizes *r* were determined ($r=Z/\sqrt{n}$; *r* < 0.1; weak, 0.1 ≤ *r* < 0.3; mean, *r* > 0.5; large). To evaluate the kinetics of dynamic measurements, the curves were parted in an exponential and a linear part describing the rapid uptake of the tracer and the much slower decay, respectively. Logarithmic or linear regression was used to assess correlation. Correlation coefficient R was calculated and therefore │R│ < 0.1, slight correlation; 0.1 ≤ │R│ < 0.3, moderate correlation; and │R│ > 0.5, strong correlation. Determination coefficient *R*^2^ represents a measurement for the goodness-of-fit and was used for the regression lines in Fig. 3. The significance levels of the measurements of the blood plasma samples were calculated using the Kruskal–Wallis test for independent samples with Bonferroni correction.

## Results

Fourteen animals were studied in WT group, and 13 animals in the TLR2^−/−^ group. Due to technical or medical complications or unsuccessful CPR, 4 animals in the WT group and 3 animals in the TLR2^−/−^ group had to be excluded. Accordingly, in each group, 10 animals could be involved in data analysis. Hemodynamic, as well as procedural data of CPR and PET-scans, are given in Table 1 and did not differ between both groups.

### Increased Uptake of Glucose in WT Mice Post-cardiac Arrest and Cardiopulmonary Resuscitation

**Fig. 2.**
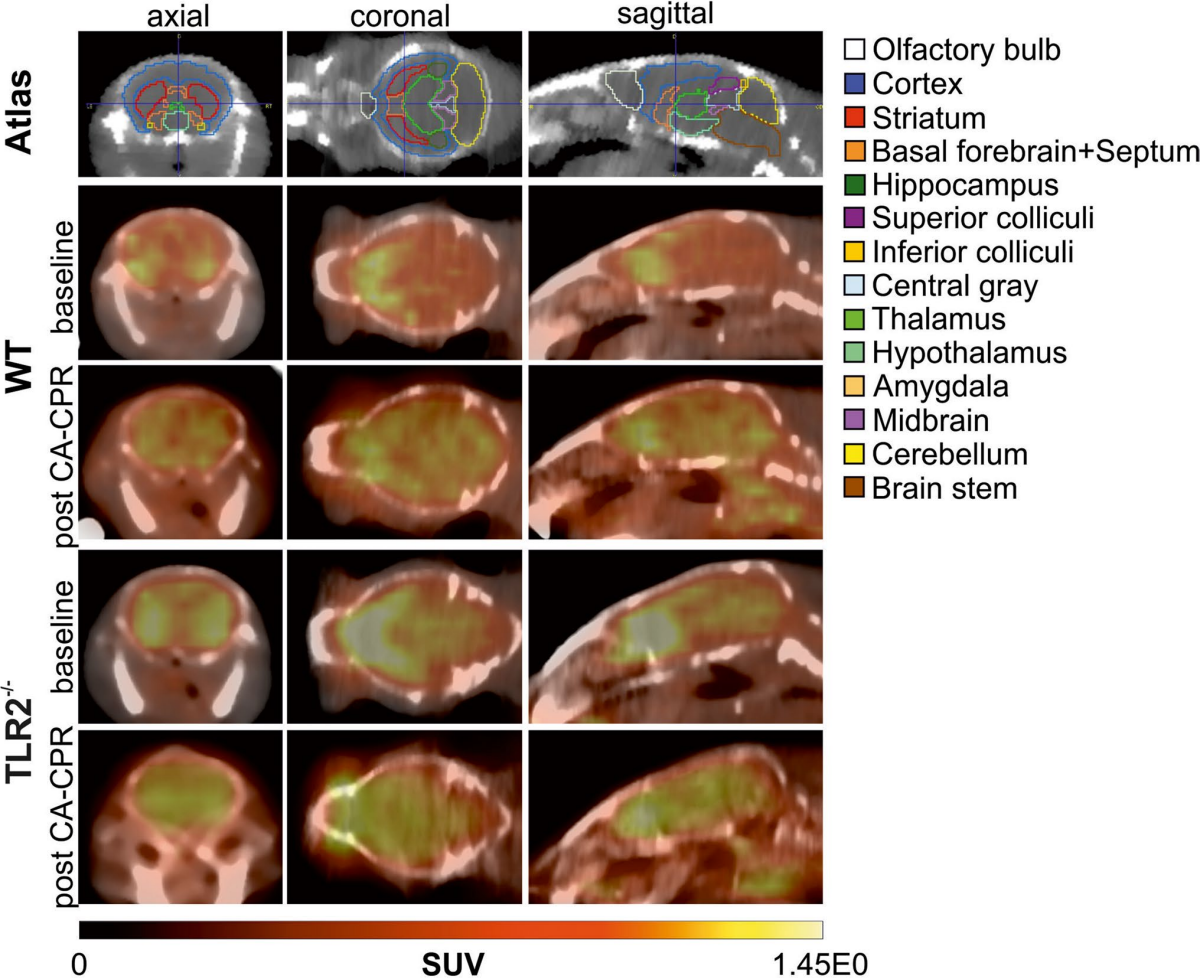
Representative examples of [^18^F]F-FDG uptake images taken between 50 and 60 min after injection of tracer in WT- and TLR2^−/−^-mouse brain baseline and post CA-CPR.

**Fig. 3.**
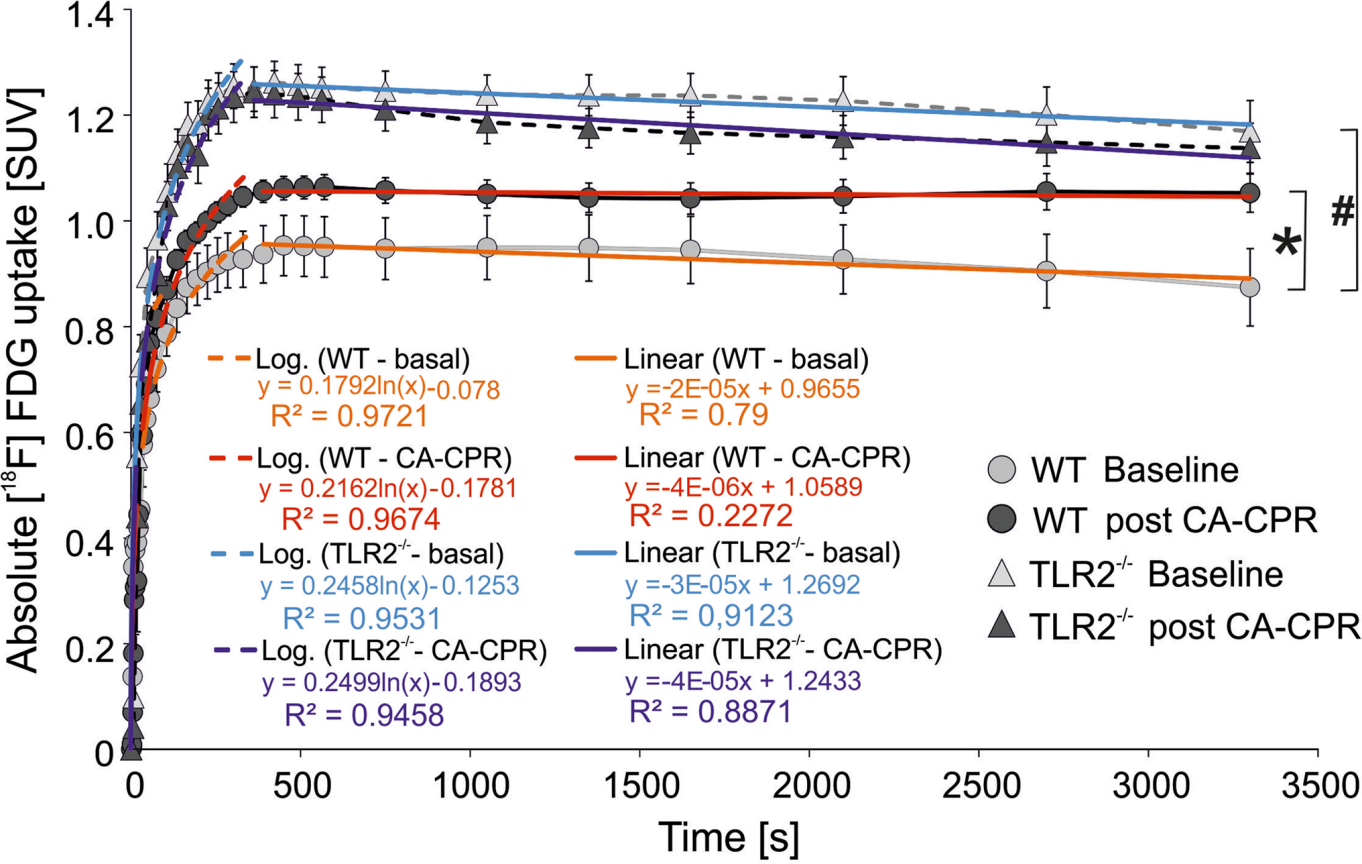
Total cerebral uptake of [^18^F]F-FDG measured in PET-CT for 1 h. Absolute [^18^F]F-FDG uptake (SUV_mean_) in WT- and TLR2^−/−^ mouse brains were measured at baseline and post CA-CPR and presented during 1 h of measurement.

Absolute uptake values determined in PET-CT images analysis (Fig. 2) showed an increase in glucose uptake over time (Fig. 3). Data showed an exponential increase at the first 400 s of all measurements. In this part, the kinetics were almost the same, supported by a strong positive correlation R between baseline and PET scans post CA-CPR (WT, *R* = 0.986; TLR2^−/−^, *R* = 0.996). As well, no difference appeared in the kinetics of exponential glucose uptake in baseline PET scans over time among the mouse strains seen by a strong positive correlation (WT vs. TLR2^−/−^, *R* = 0.969). After the saturation of glucose uptake, the curve followed a linear course with a slight slope compared to the maxima (Fig. 3). The correlation coefficients displayed a strong positive correlation within the TLR2^−/−^ animal group between baseline and PET scans post CA-CPR (*R* = 0.845), and in comparison with the baseline PET scans (WT vs. TLR2^−/−^, *R* = 0.961). In the WT animals, we found a moderate positive correlation in the curve progression (baseline vs. post CA-CPR, *R* = 0.162). The analysis of correlation shows that glucose uptake followed very similar kinetics over time in investigated groups and baseline and PET scans post CA-CPR.

**Fig. 4.**
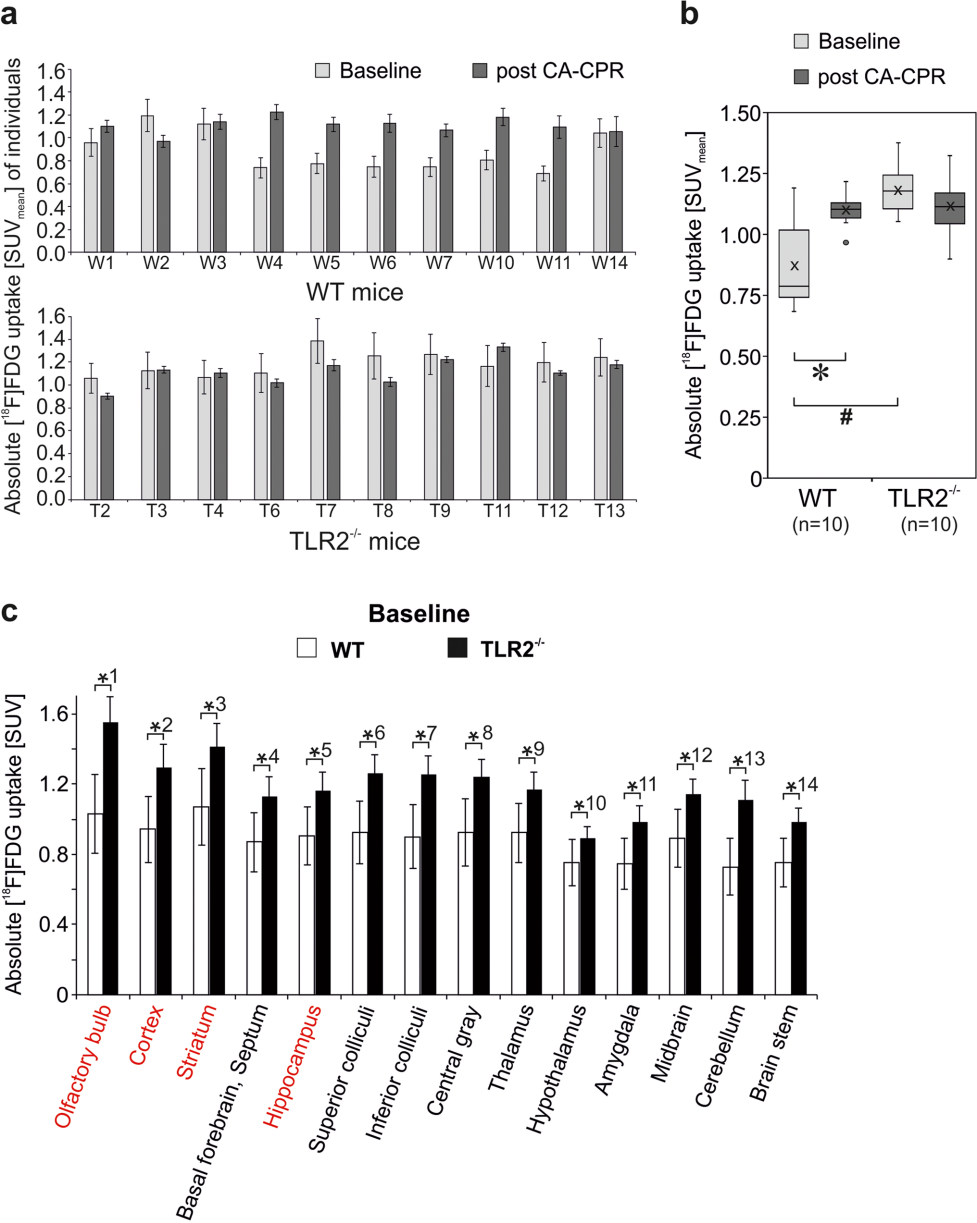
Global cerebral metabolism measured using [^18^F]FDG-PET-CT and baseline regional [^18^F]F-FDG uptake in WT and TLR2^−/−^ mice in absolute SUV. Absolute [^18^F]F-FDG uptake (SUV_mean_) in WT- and TLR2^−/−^ mouse brain regions was measured at baseline and post CA-CPR (**a**, **b**). **a** SUV_means_ of whole brain were presented for each animal as mean ± SD. **b** SUV_means_ of whole brain for the groups of animals were shown as boxplots. * *p* value = 0.017 (Wilcoxon signed-rank test), # *p* value = 0.001 (Mann–Whitney *U* test). **c** The absolute [^18^F] FDG uptake values of baseline PET scans in WT(white)- and TLR2^−/−^ (black) mouse brain regions displayed in comparison. Data shown as SUV_mean_ ± SD; (*p* values WT vs. TLR2^−/−^ mice): *^1^ 0.000, *^2^ 0.001, *^3^ 0.002, *^4^ 0.003, *^5^ 0.005, *^6^ 0.001, *^7^ 0.001, *^8^ 0.002, *^9^ 0.009, *^10^ 0.011, *^11^ 0.002, *^12^ 0.007, *^13^ 0.000, *^14^ 0.004.

When analyzing the last 15 min of measurement and plotted as absolute [^18^F]FDG uptake, a difference in quantity of glucose uptake could be revealed (Fig. 4). In Fig. 4a, the absolute uptake of [^18^F]FDG in the whole brain of every individual animal involved in data analysis is presented at baseline and post CA-CPR. In WT animals, 6/10 displayed an increase post CA-CPR, 1/10 showed reduced SUV_means_ post CA-CPR, and 3/10 were at the same level with the SUV_means_. In contrast, the SUV_means_ of all 10 TLR2-deficient animals displayed no differences between baseline and post CA-CPR uptake values. The absolute uptake of [^18^F]FDG in the whole brain was significantly higher in the group of WT animals post CA-CPR in comparison with baseline measurements (baseline SUV_mean_, 0.882 ± 0.055 vs. post CA-CPR SUV_mean_, 1.108 ± 0.021; *n* = 10, *p* = 0.017, *r* = 0.757 (large effect size), Fig. 4b). Approximately 140 min after CA-CPR, glucose uptake in the brain was increased by 25.6%. In contrast, the absolute glucose uptake in the whole brain of TLR2^−/−^ mice was not significantly different between baseline and measurements post CA-CPR (baseline SUV_mean_, 1.187 ± 0.031 vs. post CA-CPR SUV_mean_, 1.120 ± 0.036; *n* = 10, *p* = 0.114, Fig. 4b). Hence, the different mouse strains presented a different pattern in glucose uptake at baseline and post-CPR.

### Baseline Brain Glucose Uptake Is More Intense in TLR2^−/−^ than in WT Mice

In comparison, baseline measurements of both mouse strains, WT vs. TLR2^−/−^, show a highly significant difference with regard to the absolute glucose uptake in the whole brain (*n* = 10, *p* = 0.001, *r* = 0.71 (large effect size)). Baseline mean glucose uptake values of WT mice were SUV_mean_ 0.882 ± 0.055, and in TLR2^−/−^ mice, the glucose uptake displayed with SUV_mean_ 1.187 ± 0.031 was about 34.6% higher (Fig. 4b). Furthermore, the differences in the brain regions in absolute [^18^F]F-FDG uptake values between the animal strains in baseline PET scans were investigated and are given in Fig. 4c. The higher absolute SUV of glucose in TLR2^−/−^ mice extended in baseline measurements significantly over all brain regions in comparison to the WT mice (Fig. 4c). The *p* values range between *p* < 0.001 for olfactory bulb as well as cerebellum and *p* = 0.011 for the hypothalamus (Fig. 4c).

### Pattern of Glucose Uptake in Various Brain Regions Differs Between WT and TLR2-Deficient Mice After Cardiac Arrest and Cardiopulmonary Resuscitation

**Fig. 5.**
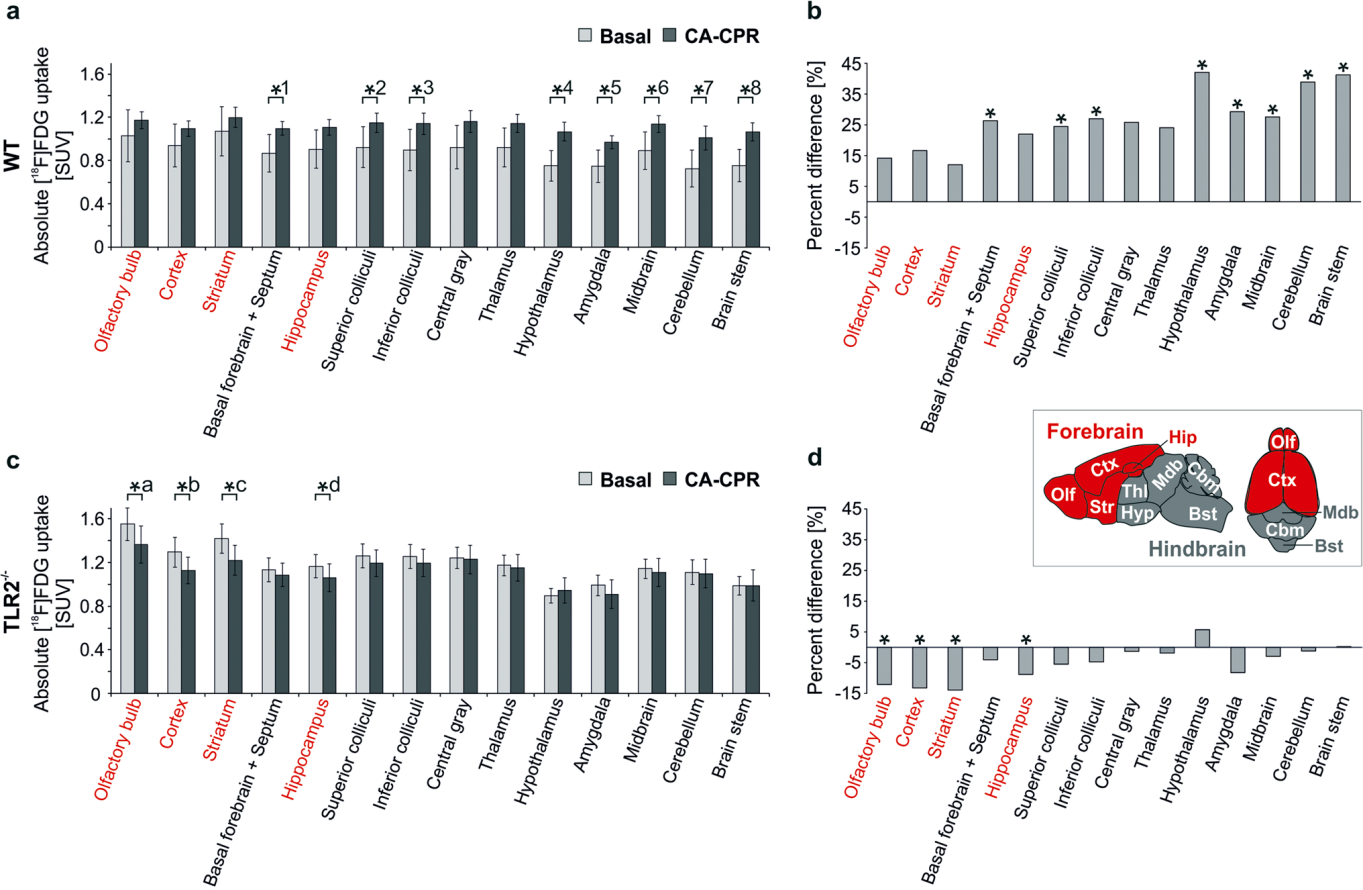
Absolute regional cerebral metabolism measured using [^18^F]FDG-PET-CT. **a** and **c** Absolute [^18^F]F-FDG uptake (SUV) in WT (**a**) and TLR2^−/−^ (**c**) mouse brains were measured at baseline and post CA-CPR. Data shown as SUV_mean_ ± SD. **a** *p* values for WT (Wilcoxon rank-sum test): *^1^ 0.017, *^2^ 0.017, *^3^ 0.017, *^4^ 0.007, *^5^ 0.017, *^6^ 0.022, *^7^ 0.013, *^8^ 0.013. Because of Bonferroni correction hippocampus (*p* = 0.037), central gray (*p* = 0.028), and thalamus (*p* = 0.028) failed significance. **c** *p* values for TLR2^−/−^ mice (Wilcoxon rank-sum test): *^a^ 0.011, *^b^ 0.011, *^c^ 0.011, *^d^ 0.021. Because of Bonferroni correction superior colliculi (*p* = 0.038) failed significance. **b** and **d** Percentage variation of absolute [^18^F]F-FDG uptake in baseline and post CA-CPR measurements in WT (**b**) and TLR2^−/−^ (**d**) mice. Inset: Schematic representation of mouse brain regions subdivided in forebrain (red) and hindbrain (grey) (after 57): Bst brain stem, Cbm cerebellum, Ctx cortex, Hip hippocampus, Hyp hypothalamus, Mdb midbrain, Ofl olfactory bulb, Str striatum, Thl thalamus.

In the WT animals, the absolute glucose uptake was increased post CA-CPR (Fig. 5a) in every brain region. Significant uptake increase was found in the basal forebrain, superior colliculi, inferior colliculi, hypothalamus, amygdala, midbrain, cerebellum, and brain stem. The increase of the SUV in the olfactory bulb, cortex, and striatum was not significant with a difference under 17%. The overall increase varied from 12.02% in the striatum and 42.04% in the hypothalamus (Fig. 5b).

Unlike in the group of TLR2^−/−^ mice, by trend, a decreased uptake of glucose post CA-CPR compared to the baseline measurements was noted. Significant differences were found in the olfactory bulb, cortex, striatum, and hippocampus (Fig. 5c). The decrease of glucose uptake was not significant with a difference of less than 9%, ranging from 13.91 (striatum) to − 1.22% (cerebellum).

When looking at the results of absolute data of investigated animal groups, apart from the fact that WT mice generally exhibited an increase and TLR2^−/−^ a decrease of glucose uptake, the regions that were significantly different when comparing baseline and measurements post CA-CPR were partly opposite (Fig. 5a and c). Whereas in TLR2^−/−^ mice, significant differences in forebrain occurred (see Fig. 5 red text and insert); this was not the case in the same brain areas of WT animals. In the WT group, significant differences were detected in the posterior cortical areas (= hindbrain; Fig. 5, insert).

### Plasma Glucose in WT and TLR2^−/−^ Mice After CA-CPR and PET-CT at the Same Level

Plasma glucose levels after completing the second PET-CT post CA-CPR were not different between WT mice and TLR2^−/−^ mice (*p* = 0.505; Table 1). For the native control samples, the plasma glucose was determined at 10.18 ± 3.6 mmol/l, ranging in the same as Bascuñana et al. [39] described. This tends to be lower compared to the mean of plasma glucose of the WT mice after CA-CPR and PET-CT (15.79 ± 7.29 mmol/l; see Table 1), but the difference was not significant (*p* = 0.6).

### Cytokines and VEGF-A in WT and TLR2^−/−^ Mice After CA-CPR and PET-CT

**Fig. 6.**
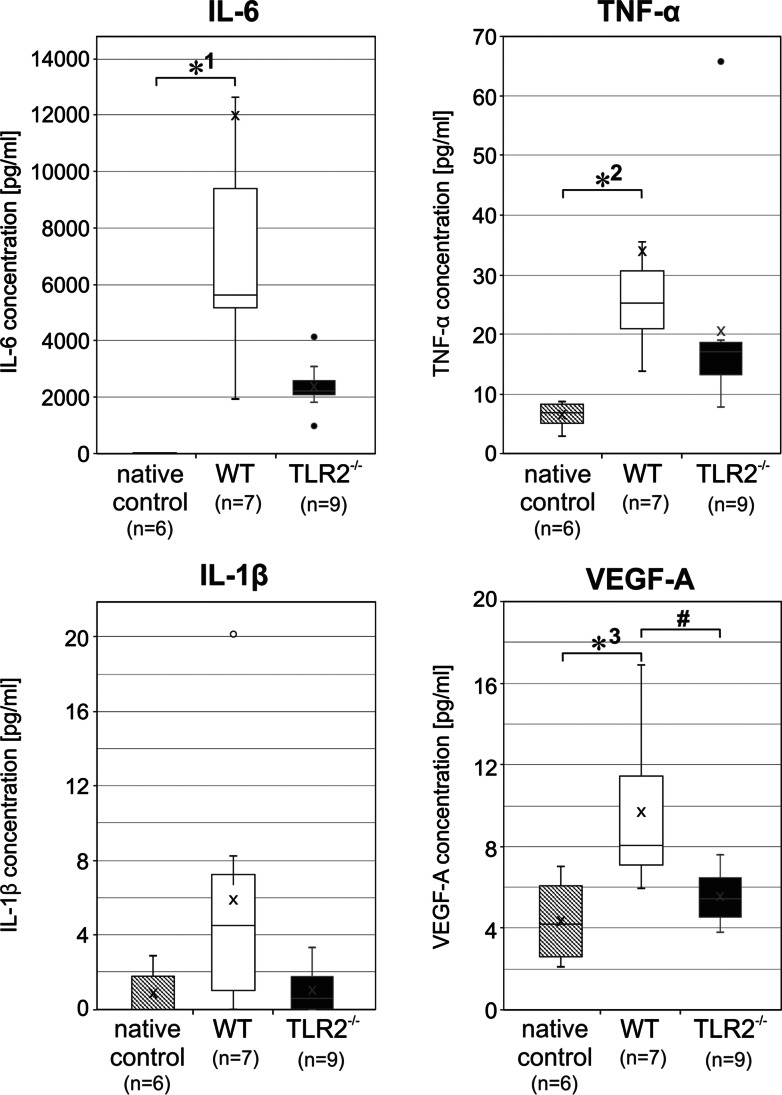
Cytokines and signal molecule VEGF-A in WT and TLR2^−/−^ mice post CA-CPR and PET-CT. Boxplots showing the quartiles, the 5th and 95th percentiles (whiskers), the median (line), and the mean (x). IL-6, the values for the native controls (0.99 ± 1,52 [pg/ml]) are not visible in the plot. *1 *p* = 0.0001; TNF-α, *2 *p* = 0.001; IL-1β, *p* = 0.097; VEGF-A, *3 *p* = 0.008; # *p* = 0.034.

Immune factors were quantified after completion of PET-CT in resuscitated mice, WT and TLR2^−/−^, approximately 3 h after induction of CA, and also in native controls. In trend, cytokine levels of IL-6, Il-1β, and TNF-α were elevated in WT mice compared to the TLR2-deficient mice. However, these values do not reach significance (Fig. 6). In contrast, comparing the levels of WT mice with the native controls, IL-6 and TNF-a were significantly increased (Fig. 6). IL-1β has shown the same trend, but failed significance with *p* = 0.073 (Mann–Whitney *U* test, Bonferroni corrected).

VEGF-A signaling molecule was increased in WT mice compared with TLR2^−/−^ mice as well as native controls (Fig. 6).

## Discussion

The present study strengthens the evidence that cerebral glucose uptake increases in the early phase after CA and CPR, and that this effect can be assessed by [^18^F]F-FDG PET. Our data is the first that quantifies this effect at the early phase after CPR in a murine model using the baseline measurement as control. In addition to general differences between the groups and pre- and post-intervention, the inter-individual differences were presented. Furthermore, this study primarily shows that this effect of enhanced glucose uptake is extenuated in TLR2-deficient individuals.

### Enhanced Absolute Glucose Uptake in the Brain of WT Mice Early After CA-CPR

It is widely agreed that the acute phase of cerebral hypoxic injury produces a marked depression of cerebral glucose metabolism that persists for several hours in various species [32, 38, 39]. Decreased glucose consumption could be explained by neuronal cell damage and following insufficiency or loss of transmitting impulses in neuronal cells [26]. In contrast to descriptions of decreasing brain metabolism after CA-CPR, in WT mice, we found enhanced absolute glucose uptake early after CA-CPR for all brain regions and this increase reached significance in the hindbrain. Despite this finding, our study indicated that the post CA-CPR [^18^F]F-FDG uptake in the whole brain of WT mice is individually different since 4/10 mice did not showed an increased glucose uptake. Investigations done with a comparable mouse model of CA-CPR also discovered significant increases in [^18^F]F-FDG uptake in the brain of mice 72 h post-CA [33]. Zhang et al. [33] chose with 72 h a considerably later time point for [^18^F]F-FDG-PET imaging, which possibly reflects progression of late brain injury. They speculate whether mitochondrial respiration is suppressed by CA-induced brain injury in the used animal model [33]. The results could therefore reflect an increase in glucose uptake due to a switch of glucose metabolism from oxidative respiration to glycolysis, similar to the Warburg effect in cancer [33]. It is obvious to assume that this switch of glucose metabolism occurred at an early time point after CA, but has yet to be proven. Although the etiology of increased glucose metabolism is unclear and some earlier studies show an impaired glucose metabolism after cardiac arrest ([^18^F]F-FDG PET, canine 39; [^18^F]F-FDG PET and autoradiographic images analysis, rats 32; gerbil 40; biochemical analysis, rats 41), an increased glucose metabolism can be also explained by the upregulated inflammatory response initiated with hypoxic impairment. For several immune cells including macrophages, T cells, and neutrophils, in particular, if they are activated, elevated glycolysis has been described. Activated and infiltrating inflammatory cells utilize glucose at a much higher level than peripheral non-inflammatory cells [,^27^, ^42^,^43^]. An early increase in [^18^F]F-FDG uptake in the whole brain with high significance in cortex and cerebellum was observed 5 h following the induction of systemic inflammation like sepsis-associated encephalopathy (SAE) in mice [31]. In vitro measurements with autoradiography imaging after lipopolysaccharide stimulation in mice also presented enhanced [^18^F]F-FDG uptake in spleen and lymph nodes, because B cells increase their glucose consumption very early after TLR treatment [44]. Such findings could explain the rise in [^18^F]F-FDG uptake we measured and make them the most likely cause of the increase as CA-CPR induces cerebral inflammatory processes. Hosmann et al. [45] established a microdialysis setting in vivo to investigate the impact of resuscitation methods on cerebral and peripheral metabolism for lactate, glucose, and glutamate simultaneously. Their results that cerebral glucose levels fell below the detection limit during CA-CPR, returned to baseline level after ROSC, and are significantly elevated 16 to 48 min after ROSC support our findings [45]. As previously mentioned, Zhang and colleagues discovered a rise in glucose uptake 72 h post-CA with significance in the hindbrain; in contrast, the increase in the regions of the forebrain failed to reach significance [33] corresponding to our results presented for the immediate period after ROSC.

### TLR2^−/−^ Mice Showed No Difference Between Baseline Brain Glucose Uptake and Early After CA-CPR But Displayed Higher Glucose Level than WT Mice

In WT mice, an increase of [^18^F]F-FDG uptake post CA-CPR was observed, whereas in TLR2^−/−^ mice, no difference of [^18^F]F-FDG uptake between baseline and post CA-CPR measurements was detected. As described for rats, the cerebral glucose levels also possibly fall during CA-CPR in TLR2^−/−^ mice and return to normal after ROSC [45], but do not rise immediately after ROSC. Maybe at this early point in time when the PET scans were done, the lack of TLR2, one of the key innate immune sensors, restricted the signaling of early immune response that usually occur under I/R damage. The TLR2^−/−^ mouse strain has shown a reduced increase in immune response 8 h following CA-CPR as well [24]. Hence, the mouse strains used for the study presented completely different pattern of glucose uptake in the brain at baseline and post CA-CPR, although the blood glucose level was the same at the end of PET-CT post CA-CPR. An earlier study done with the same mouse strains and the same animal model of CA-CPR described a different increase of plasma levels between WT and TLR2^−/−^ mice 8 h following CA-CPR such as IL-6 in WT group was increased about 100-fold, whereas in TLR2^−/−^ group, the increase was only 5 times [24]. Our results of cytokines in blood plasma 3 h following CA-CPR also suggest these differences in the increase but did not reach significance. This may be due to the early time of measurement. These differences in the increase of inflammation markers in the mouse strains used support the assumption that elevated inflammation raises the need for glucose. In addition to a reduced increase of inflammation markers, Bergt et al. [24] revealed evidence that a lack or inhibition of TLR2 signaling is associated with improved survival and upgraded preservation of motor function and cognitive capacity following CA-CPR. Activation of TLR2 initiate the transcription of genes associated with innate immune responses and inflammation, which leads to tissue injury by the initiation of apoptotic pathways also in the brain [19]. So, the lack of TLR2 could explain the equal levels of glucose uptake at baseline and post CA-CPR in the brain, because of the significant lower inflammatory reaction in TLR2^−/−^ mice after CA-CPR [24]. VEGF-A is a potent angiogenic factor and it has the ability to induce transient vascular leakage [46]. I/R have been shown to be relevant stimuli for rapid VEGF expression [47, 48]. In our study, plasma protein level of VEGF-A was significantly increased in WT mice within 3 h after ROSC. VEGF serum protein levels were also increased after ROSC in a swine model of CA-CPR [49]. There is increasing evidence that the expression of VEGF is elevated following activation of TLR2 [50, 51]. Induction of VEGF after CA-CPR possibly causes increased vascular permeability and thus blood–brain barrier opening [52] leading to a vasogenic edema. VEGF-induced brain stem edema may be the primary cause of the increased mortality seen in the first 3 days after successful resuscitation from CA. The not elevated plasma VEGF-A in the TLR2^−/−^ mice in our study could give evidence for the better survival of this mice [24]. Information on the role of TLR2 deficiency in cerebral I/R injury are varying. In addition to inflammatory signals leading to tissue injury, TLR2 induces also protective signals that result in production of cytoprotective molecules such as heat shock proteins and Bcl-2, an anti-apoptotic molecule [53]. There are suggestions that activation of innate immunity in the brain is an ambiguous event that can be advantageous or disadvantageous for the fate of the host depending on the specific conditions of neuronal injury and the balance between inflammatory and protective signals [54].

If we consider the results of baseline glucose uptake and post CA-CPR, the different patterns of glucose uptake detected amongst the mouse strains support the important well-known role of TLR2 in inflammatory reaction and now extend to a potential cross-link to energy metabolism. These results could lead to additionally using glucose metabolism as a method for predicting the course of inflammation in post-cardiac arrest syndrome.

### Higher Baseline Glucose Level in TLR2^−/−^ Mice

In our study, the baseline absolute brain glucose uptake values in the TLR2^−/−^ mice were much higher than in WT mice. To address these differences in the baseline glucose metabolism, further investigations for characterizing the normative profile of WT and TLR2^−/−^ mice have to be done. In TLR2^−/−^ mice fed a high-fat diet, body weight gain was significantly less compared with WT mice [55]. The high-fat diet–induced increases in fasting blood glucose levels, as well as in circulating insulin and leptin levels, were absent in TLR2^−/−^ mice. High-fat feeding induced increases in overall fat mass, and in fat mass of different pockets were abrogated in TLR2^−/−^ mice [55]. Therefore, we speculate that the TLR2^−/−^ mice have altered metabolism and different baseline glucose levels due to knockout. On this assumption, it also seems possible that the higher glucose uptake in WT mice could be due, in part or in whole, to the switch to glycolysis after hypoxia, although both mouse strains were subjected to exactly the same ischemic insult.

However, our present study has some limitations. First, the use of anesthesia during the experimental procedure may have affected the distribution of brain glucose metabolism [38], as anesthesia, especially the use of ketamine/xylazine, is known to reduce metabolism throughout the murine brain and result in a lower uptake of [^18^F]F-FDG compared to isoflurane or awake [38]. At the same time, ketamine/xylazine anesthesia generate increased blood glucose levels [38], due to a known pharmacological effect of xylazine. Because of the experimental setting in the present study, it has to be taken into consideration that all baseline PET scan recordings were started after induction of anesthesia, tail vein catheter, and bedding, whereas PET scans following CA-CPR were started about 2 h after induction of anesthesia. At that time, the effect of anesthesia was decreased and the mice were at rest and still unconscious. Second, after CPR, only one time point was studied. We were interested in mechanisms of the early phase after global ischemic insult because this is the phase for diagnostics and concentrated efforts to find the optimal treatment strategy. Third, furthermore, FDG-PET provides information about the uptake of glucose and the first part of glycolysis but does not evaluate glucose metabolism beyond this [56].

## Conclusion

We found that cardiac arrest and resuscitation induced a different glucose metabolism post-ischemia associated with the function of key immune factor TLR2 compared to the basal grade of glucose metabolism. Upcoming experiments will be focused on the different baseline glucose uptake in TLR2-deficient mice and what influence do an elevated glucose metabolism have on the post-cardiac arrest syndrome and outcome. PET targeting brain metabolism and further development of new tracers provide new tools to track the progression of diseases and may help uncover the pathophysiological changes in the brain after ischemic injury. More studies on PET-based predictions on the outcome are needed.
